# Fibre Intake Is Independently Associated with Increased Circulating Interleukin-22 in Individuals with Metabolic Syndrome

**DOI:** 10.3390/nu11040815

**Published:** 2019-04-11

**Authors:** Luciana Torquati, Jeff S. Coombes, Lydia Murray, Sumaira Z. Hasnain, Alistair R. Mallard, Michael A. McGuckin, Robert G. Fassett, Ilaria Croci, Joyce S. Ramos

**Affiliations:** 1 School of Human Movement and Nutrition Sciences, The University of Queensland, Brisbane, Queensland 4072, Australia; jcoombes@uq.edu.au (J.S.C.); a.mallard@uq.edu.au (A.R.M.); r.fassett@uq.edu.au (R.G.F.); ilaria.croci@ntnu.no (I.C.); joyce.ramos@flinders.edu.au (J.S.R.); 2 School of Sport and Health Sciences, University of Exeter, Exeter EX4 4PY, UK; 3 Inflammatory Disease Biology and Therapeutics Group/Immunopathology Group, Translational Research Institute, The University of Queensland, Brisbane, Queensland 4072, Australia; lydiasorayamurray@gmail.com (L.M.); sumaira.hasnain@mater.uq.edu.au (S.Z.H.); michael.mcguckin@unimelb.edu.au (M.A.M.); 4 Australian Infectious Disease Research Centre, The University of Queensland, Brisbane, Queensland 4072, Australia; 5 Faculty of Medicine, Dentistry and Health Science, The University of Melbourne, Parkville, Victoria 3010, Australia; 6 K.G. Jebsen Center of Exercise in Medicine, Department of Circulation and Medical Imaging, Faculty of Medicine, Norwegian University of Science and Technology, 7491 Trondheim, Norway; 7 SHAPE Research Centre, Exercise Science and Clinical Exercise Physiology, College of Nursing and Health Sciences, Flinders University, Bedford Park, South Australia 5042, Australia

**Keywords:** dietary fibre, immune response, metabolic endotoxemia

## Abstract

The positive effects of dietary fibre on gut barrier function and inflammation have not been completely elucidated. Mice studies show gut barrier disruption and diet-induced insulin resistance can be alleviated by cytokine interleukin-22 (IL-22). However, little is known about IL-22 in humans and its association with gut-beneficial nutrients like fibre. We investigated whether fibre intake was associated with circulating levels of IL-22 in 48 participants with metabolic syndrome (MetS). Bivariate analysis was used to explore associations between circulating IL-22, fibre intake, MetS factors, body composition, and cardiorespiratory fitness (peak oxygen uptake, $\dot{V}$O_2peak_). Hierarchical multiple regression (HMR) was used to test the independent association of fibre intake with circulating IL-22, adjusting for variables correlated with IL-22. Circulating IL-22 was positively associated with fibre intake (r_s_ = 0.393, *p* < 0.006). The HMR-adjusted model explained 40% of circulating IL-22 variability, and fibre intake significantly improved the prediction model by 8.4% (*p* < 0.022). Participants with fibre intake above median intake of 21.5 g/day had a significantly higher circulating IL-22 than the lower intake group (308.3 ± 454.4 vs. 69.0 ± 106.4 pg/mL, *p* < 0.019). Fibre intake is independently associated with increased circulating IL-22 in individuals with MetS. Findings warrant further investigations to evaluate whether changes in dietary fibre intake alter circulating IL-22, and its effects on health outcomes.

## 1. Introduction

Interleukin-22 (IL-22) is a cytokine of the IL-10 family that is produced by leukocytes and acts on non-leukocytes, particularly in epithelial tissues and other organs such as the liver and pancreas, where it is involved in promoting barrier integrity and wound repair [1]. Disrupted epithelial barriers can lead to systemic inflammation and altered metabolic signalling [2]. Thus, factors promoting gut barrier integrity may protect against systemic disorders involving inflammation such as metabolic syndrome (MetS). Like IL-22, dietary fibre has been associated with both promoting gut barrier heath and ameliorating MetS and type 2 diabetes (T2D) by modulating the intestinal microbiota and its influence on the epithelium and underlying immunity [1,3]. However, it is not known whether fibre intake alters mucosal immune cell IL-22 production, nor whether IL-22 production is associated with the level of fibre in the diet in individuals with MetS.

Dietary fibre is fermented in the gut and their products (including short chain fatty acids, SCFAs) exert an anti-inflammatory effect on gut epithelial cells and on immunoregulatory cells such as macrophages, dendritic cells, effector T cells, and innate lymphoid cells, which can in turn produce IL-22 [4,5,6,7]. In mice, a high-fibre diet was shown to stimulate SCFA expression and consequently release IL-18, which is also involved in epithelial repair. In a study by Macia et al. (2015) using an induced-colitis model, epithelial damage was inversely proportional to the amount of fibre in the diet [8]. While this suggests fibre’s protective role on the gut could be mediated by the release of cytokines such as IL-22, studies on IL-22 and diet are limited to murine models and to the negative effects of high-fat diet [8,9]. Little is known about the association of IL-22 with potential beneficial nutrients, like dietary fibre.

Studies in mice have suggested a role for IL-22 in MetS and T2D treatment, as IL-22 administration resulted in improved glycaemic control, insulin sensitivity, and lipid metabolism, concomitantly with improved intestinal barrier function and reduced inflammation [1,3,9,10,11]. However, evidence in humans is limited and conflicting. Cross-sectional studies reported higher plasma IL-22 concentrations in individuals with T2D compared to both lean and obese healthy controls [12,13,14]. Likewise, increased circulating IL-22 was associated with being male, smoking, lower glomerular filtration rate, T2D, and cardiovascular disease [15,16]. Human studies have reported on the associations of IL-22 with obesity, T2D, and cardiovascular disease, but none of these investigated dietary factors or described such associations in people with MetS.

Thus, the aim of this study was to examine the independent association of fibre intake and circulating IL-22 in participants enrolled in the Exercise in the prevention of Metabolic Syndrome (EX-MET) study. We hypothesized an independent positive association of fibre intake and circulating IL-22 in this population.

## 2. Materials and Methods

Materials and Methods. This is a sub-study of baseline data from participants enrolled in the Exercise in the prevention of Metabolic Syndrome (EX-MET) study [17]. The EX-MET study included only participants with metabolic syndrome, as defined by the International Diabetes Federation criteria [18] and was approved by the Medical Research Ethics Committee, The University of Queensland, Brisbane, Australia (2012000627). Participants in the EX-MET study were excluded if any of the following criteria were present: unstable angina, recent myocardial infarction (4 weeks), severe valvular heart disease, uncompensated heart failure, pulmonary disease, uncontrolled hypertension, kidney failure, and cardiomyopathy. All participants enrolled in EX-MET study provided oral and written informed consent. Participants were included in the current sub-study if they consented to provide blood samples for immunological markers’ analysis and their serum samples were available (74% of enrolled participants).

As part of this study, demographic information and lifestyle measures were taken including smoking status and dietary intake. Clinical measures in this study included (1) lipid profile (total cholesterol, triglycerides, high-density lipoprotein cholesterol (HDL-C) and low-density lipoprotein cholesterol (LDL-C)); (2) inflammatory markers (hsCRP, IL-22); (3) glucose metabolism markers (fasting glucose, insulin, HbA1c; and C-peptide; glucose and insulin concentrations); (4) body composition (fat and lean body mass percentage, weight, waist and hip circumferences); and (5) cardiorespiratory fitness (peak oxygen uptake, $\dot{V}$O_2peak_).

Dietary fibre intake was assessed using a 3-day food diary. Participants were instructed to record any food and drinks consumed in the past 72 h prior to baseline measurements (blood samples and other metabolic measures as described in Ramos et al [17]). The diary was collected by a research assistant, who then conducted a short interview with the participant to check the accuracy of the recall. Data was entered and analysed in FoodWorks (Xyris Software, Spring Hill, Queensland, Australia). This version uses the AUSNUT 2011-13 and NUTTAB 2010 databases to analyse macro- and micronutrient compositions. Total dietary fibre intake was calculated and expressed in grams per day.

Following a 12-h overnight fast, blood samples were withdrawn from an ante-cubital vein. Samples were collected into tubes with and without anticoagulants to acquire plasma and serum samples, respectively. These were then stored at −80 °C and later used to analyse circulating IL-22, HsCRP, insulin, C-peptide, and intact proinsulin. Circulating IL-22 was measured from serum samples via ELISA MAX^TM^ Deluxe Set (Biolegend, 434504, San Diego, CA, USA) according to manufacturer’s instructions. Briefly, the plates were coated with the capture antibody at 4 °C for 24 h, and were subsequently washed with phosphate-based saline (PBS) 0.05% Tween 20 and non-specific binding was blocked using 1% bovine serum albumin in PBS (pH 7.4) at room temperature for 2 h, shaking at 500 rpm. Serum samples (1:100 dilution) then coated the plates for up to 3 h, before being detected by detection antibodies (Avidin-HRP and TMB substrates). Within 15 min, absorbance was read at 570 nm and 450 nm (data presented as Absorbance_570 nm–450 nm_ against the standard curve for hIL-22 provided). An ELISA kit was also used to measure HsCRP (K-ASSAY High-Sensitive C-Reactive Protein kit KAI-160, Kamiya Biomedical, Seattle, WA, USA) and intact proinsulin (human intact proinsulin ELISA, EZHIPI-17K, Merck Millipore, Darmstadt, Germany), according to the manufacturer’s instructions. C-peptide and insulin concentrations were analysed using electrochemiluminescence immunoassay (ECLIA) via Cobas e411 immunoassay analyser (Roche Diagnostics, Indianapolis, IN, USA). HbA1c was also derived from whole blood samples (Rx Daytona Plus, Randox Laboratories, Crumlin, Antrim, UK). Using the measured C-peptide and insulin values, “proinsulin to insulin” and “proinsulin to C-peptide” ratios were calculated. Those samples with proinsulin values below detection threshold (0.5–100 pM) were assigned a value of 0.1 pM for statistical analysis.

Fasting glucose and lipid profile (total cholesterol, high-density lipoprotein cholesterol (HDL-C), low-density lipoprotein cholesterol (LDL-C), and triglycerides) values were also measured after a 12-h fast via a finger prick sample, which was subsequently analysed using the Cholestech LDX system. Homeostasis model assessment for insulin resistance (HOMA-IR) and insulin secretion (HOMA-B) values were calculated via HOMA2 calculator version 2.2 (University of Oxford) [19,20].

Weight, waist circumference and blood pressure (BP) were measured as described in previously [21]. Those individuals either on hypertensive medication or with systolic BP ≥ 140 and diastolic BP ≥ 90 were classified as hypertensive [22]. Body composition was measured with dual-energy X-ray absorptiometry (DEXA, Hologic QDR4500A version12.5, Waltham, MA, USA), and cardiorespiratory fitness was assessed during a graded exercise test, measured via indirect calorimetry (Metamax II, Cortex, Leipzig, Germany; or Parvo Medica TrueOne 2400 system, ParvoMedics Inc., Sandy, UT, USA) on either a treadmill or cycling ergometer depending on the individual’s physical capability [14]. Briefly, the test protocol started with a familiarisation phase consisting of two 4-min warm up stages (4 km/h at 0% incline or 50–60 rpm at 0 W, and 4 km/h 4% incline or 50–60 rpm at 25 W). Then, the test started at a higher intensity (6% incline or 50 W) with subsequent increases in intensity (2% incline and 25 W increases) every minute until exhaustion. Cardiorespiratory fitness was determined as the peak oxygen consumed ($\dot{V}$O_2peak_) during the graded exercise test and was expressed as absolute (L/min) and relative to fat-free mass (mL/kg_FFM_/min) to eliminate adiposity as a confounding factor.

### Statistical Analysis

Bivariate correlations were used to explore associations between circulating IL-22 and demographic measures, fibre intake and macro nutrients, MetS factors (waist circumference, BP, glucose metabolism markers, lipid profile markers), MetS severity (sex-specific *z*-scores calculated using the formula described in Malin et al. [23]), inflammatory markers (HsCRP), body composition, and cardiorespiratory fitness. A Chi-squared test was used to test associations among categorical variables. Variables were checked for normality and those not normally distributed were log transformed. For those variables not normally distributed even after log-transformation, Rho-spearman and Mann–Whitney U test (categorical) were used to explore associations. Both primary outcome measures (IL-22 and dietary fibre) were not-normally distributed, and IL-22 was still not after log-transformation. We used hierarchical multiple regression to test the independent association of fibre intake with circulating IL-22 (dependent variable). The first block (Model 1), included those variables that significantly correlated with IL-22 (waist-to-hip ratio WHR, HDL-C, smoking, relative $\dot{V}$O_2peak_, lean body mass), but excluding those variables that presented collinearity with included variables. This model also included factors that had previously been associated with circulating IL-22 levels (sex and age) [15]. In the second block of this model we included fibre intake (g/day), to assess the predictive value of fibre intake, above and beyond other factors associated with IL-22 (Model 2). An independent *t*-test was conducted to compare circulating IL-22 levels between high- and low-fibre intake groups, defined by above and below sample median intake. All analyses were performed in SPSS version 24 (IBM, New York, NY, USA).

## 3. Results

### 3.1. Participants Characteristics

Table 1 shows the characteristics of the individuals included in this study. Participants averaged 56.6 years and the majority were men and obese. About 70% of participants had 4 or more MetS factors, with more than half having hypertension and approximately 40% T2D. The median fibre intake was 21.5 g/day, which was slightly below the current recommendation of 25–30 g/day in Australia [24]. The average circulating serum IL-22 value was 153.1 pg/mL, with a large variability across participants. Glucose metabolism and lipid profile markers were slightly above reference values, as expected in this population. 

### 3.2. Fibre Intake and IL-22 Correlations

Table 2 shows the results of the bivariate correlations, using Spearman’s rho test. Circulating IL-22 was positively associated with fibre intake (r_s_ = 0.393, *p* < 0.006), carbohydrate intake (r_s_ = 0.352, *p* = 0.013), absolute cardiorespiratory fitness (r_s_ = 0.330, *p* < 0.022), waist-to-hip ratio (r_s_ = 0.389, *p* < 0.006), lean body mass (r_s_ = 0.254, *p* = 0.030), and smoking status (Chi-squared = 6.137, *p* = 0.046); while it was negatively associated with HDL-C (r_s_ = −0.378, *p* < 0.008). There were no significant associations between IL-22 and participants’ medication status (Mann–Whitney U test *p* > 0.05 for metformin, aspirin, statins, beta-blockers, calcium antagonists, angiotensin II).

**Figure 1:**
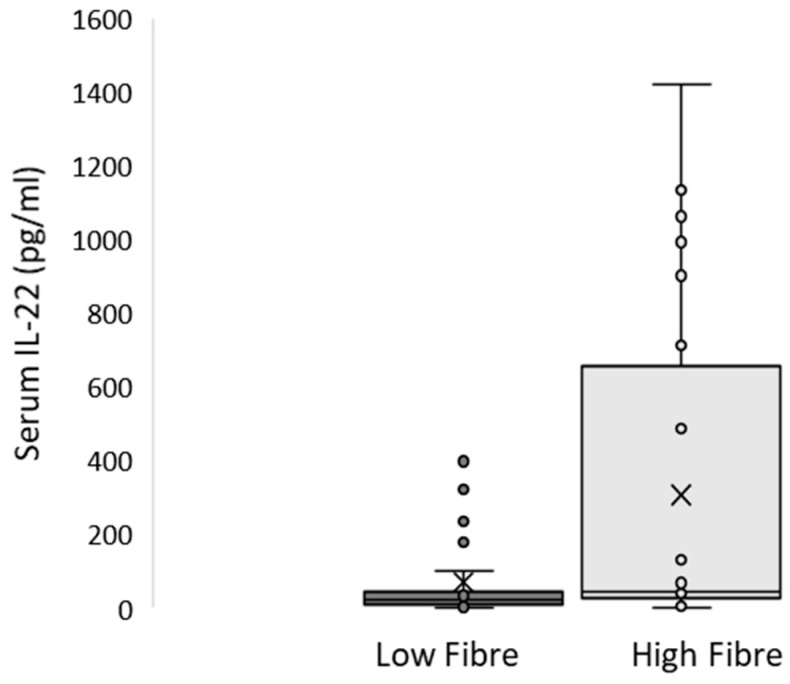
Circulating IL-22 concentration by fibre intake groups. The high-fibre intake group (above median intake of 21.5 g/day) had a significantly higher circulating IL-22 than the low-fibre intake group (mean 308.3 ± 454.4 vs. 69.0 ± 106.4 pg/mL, respectively *p* < 0.019).

The results of the hierarchical multiple regression are shown in Table 3. Models 1 and 2 explained about 34% and 42% of circulating IL-22 variability, respectively. In Model 2, fibre intake significantly improved the prediction by 8.4% (*p* < 0.022). The regression coefficient analysis showed that fibre intake remained a significant predictor of circulating IL-22 levels, even after accounting for confounding factors (Model 2 β = 0.361, *p* = 0.022). When comparing participants based on their fibre intake (above and below a median intake of 21.5 g/day), those in the high-intake group showed higher serum circulating IL-22 (see Figure 1). There was no significant difference between groups for MetS severity and other metabolic markers (see Supplement Table S1).

## 4. Discussion

To our knowledge, this is the first study to report on associations between circulating IL-22 and dietary fibre intake in humans with MetS. We found fibre intake was an independent predictor of circulating IL-22 even after accounting for multiple confounders, including anthropometric and metabolic markers, and lifestyle factors. Higher fibre intake was associated with higher circulating IL-22 levels, which could be considered an additional reason to promote fibre intake in MetS patients.

Animal studies showed that reduced IL-22 expression is associated with negative health outcomes, and that diet can impact gut environment and consequent expression of IL-22 [25]. Wang et al. [25] showed that mice fed with high-fat diet had reduced IL-22 expression, which was in turn associated with disrupted gut epithelial integrity. In a similar model, colonic epithelial cell stress was reversed by administering IL-22 [9]. These results suggest the potential positive effect of increased circulating IL-22 on health outcomes. The association between dietary fibre intake and circulating IL-22 shown in our study are consistent with a study in mice fed a high fat diet with or without fermentable fibre, which showed that fibre induced IL-22 production in the intestinal mucosa prevented the development of MetS [11]. These findings warrant further investigation of IL-22 modulation through diet and associated health outcomes in humans.

A recent comprehensive review concluded that IL-22 might have a dual effect being both pro- or anti-inflammatory depending on the context, tissue, and presence of other similar cytokines [26]. For example, increased serum IL-22 is found in asthma patients, where it seems to negatively regulate established allergic inflammation [27]. In a recent cross-sectional study, serum IL-22 concentrations were associated with cardiometabolic markers but also with inflammatory cytokines (IL-1 receptor antagonist, IL-18) [15]. Similar to the findings of Herder et al. [15], we observed a negative association between IL-22 and HDL-C levels, but found an opposite association with smoking and age, and no association with MetS severity (see Supplement Table S1 and Figure S1). The divergent findings might be explained by differences in sample characteristics, since our participants had a higher BMI and prevalence of T2D, but lower mean age and smoking prevalence. 

Altogether, studies suggest that increased expression of IL-22 might limit the collateral damage of the immune system activation and response [28]. Given that MetS origin is characterised by low-grade inflammation, higher IL-22 could be seen as a positive response to down-regulate such established inflammation. Murine studies showed that exogenously administering IL-22 reversed the obesity and high-fat diet induced endoplasmic reticulum (ER) stress and pancreatic stress [9,10]. This indicates that higher levels of IL-22 could alleviate obesity-induced insulin-resistance, an important stepping stone in the development of MetS [9]. However, as circulating IL-22 might be the result of multiple tissues expression, it is important that future studies measure local concentrations in addition to circulating IL-22. This would facilitate the identification of specific IL-22 expression patterns associated with dietary and metabolic markers. Future studies are needed to further investigate whether changes in fibre intake would be able to increase circulating (and local) IL-22, and whether such changes result in specific metabolic outcomes in both healthy and clinical populations. 

This is the first study to our knowledge that reported on the associations between circulating IL-22 and dietary fibre intake in humans. We also showed IL-22 was associated with body composition (lean body mass, WHR), and cardiorespiratory fitness. Participants were assessed comprehensively, including a large number of clinical measures such as body composition, inflammatory markers, glucose and lipid metabolism markers, and lifestyle factors. Investigating associations between these measures and circulating IL-22 contributed to the limited evidence of human studies in this area. Although the study and estimator accuracy would have benefited from a larger sample size, we were able to detect a small effect size resulting from adding fibre intake to the regression model (F^2^ = 0.15). Further, having controlled for this large number of confounding factors ensured robustness of our analysis and results. The use of 3-day dietary assessment tool provided a good estimate of usual dietary intake. Future studies should consider a combination of this tool and food-frequency questionnaires (for long term intake assessment). Our results are limited by the cross-sectional design, inclusion criteria, lack of healthy controls, and inability to measure potential mediators of fibre/IL-22 relationship, such as short chain fatty acids and gut microbiome [8]. Future studies should incorporate these to better describe the mechanism behind the fibre/IL-22 association in the context of health outcomes.

## 5. Conclusions

Fibre intake is independently associated with increased circulating IL-22 in individuals with MetS. These results provide further evidence on the importance of high dietary fibre intake. Further investigations using well-controlled trials should investigate whether changes in dietary fibre intake alter circulating IL-22, and the effects this may have on health outcomes.

## Figures and Tables

**Table 1:** Participants demographic, clinical, and lifestyle characteristics.

Variable	All ^1^
Age (years)	56.6 ± 9.5
Male %	62.5
**Clinical characteristics**	
Hypertension %	70.8
Systolic BP (mmHg)	132.2 ± 14.3
Diastolic BP (mmHg)	84.2 ± 9.4
Type 2 diabetes %	41.7
≥4 MetS factors (%)	70.0
Body composition	
Body fat %	39.1 ± 6.7
BMI (kg/m^2^)	31.7 ± 4.8
Waist circumference (cm)	103.7 ± 11.8
Waist-to-hip ratio	0.93 ± 0.1
Inflammatory markers	
IL-22 (pg/mL)	153.1 (47.0)
HsCRP (mg/L)	1.8 (2.2)
Glucose metabolism	
Fasting glucose (mmol/L)	6.6 (4.1)
Fasting insulin (pmol/L)	117.3 (38.4)
HbA1c (%)	6.3 ± 1.4
HOMA-IR	5.9 ± 4.7
HOMA-B	148.3 (13.7)
Pro-insulin (pmol/L)	4.6 ± 6.8
C-peptide (pmol/L)	1168.6 (704.1)
Lipid profile	
Total cholesterol (mmol/L)	4.8 (2.5)
HDL-C (mmol/L)	1.1 (0.6)
LDL-C (mmol/L)	2.9 (0.8)
Triglycerides(mmol/L)	2.0 (0.9)
**Lifestyle characteristics**	
Diet	
Total energy intake (kJ/day)	8665.5 (4716.2)
Carbohydrate intake (g/day–%Energy)	194.0 (92.6)–40.2 ± 10.2
Protein intake (g/day–%Energy)	104.1 (52.2)–20.3 ± 4.7
Fat intake (g/day–%Energy)	79.3 (31.3)–34.0 ± 8.7
Fibre intake (g/day)	21.5 (11.5)
Cardiorespiratory fitness ($\dot{V}$O_2peak_)	
Absolute (L/min)	2.6 ± 0.7
Relative (mL/kg_FFM_/min)	26.3 ± 6.9
Smoking (%)	2.1
Alcohol intake (drinks/week)	6.1 ± 8.9

^1^ Mean ± standard deviation, median (interquartile range) for variables not normally distributed. BP: blood pressure; BMI: body mass index; MetS: metabolic syndrome; IL-22: interleukin-22; HsCRP: high-sensitivity c-reactive protein; HbA1c: glycated haemoglobin; HOMA-IR: homeostasis model assessment for insulin resistance; HOMA-B: homeostasis model assessment for insulin secretion; HDL-C: high-density lipoprotein cholesterol; LDL-C: low-density lipoprotein cholesterol; $\dot{V}$O_2peak_: peak oxygen uptake; FFM: fat-free mass.

**Table 2:** Bivariate correlations between IL-22 and demographic, lifestyle, and clinical measures in participants diagnosed with metabolic syndrome.

Variable	Correlation Coefficient	*p*-Value
Age (years)	−0.252	0.084
Sex ^1^	3.200 ^1^	0.074
BMI (kg/m^2^)	0.018	0.901
Waist circumference (cm)	0.253	0.082
Waist-to-hip ratio	0.389	0.006
Body fat %	−0.244	0.095
Lean Body Mass (g)	0.254	0.030
Number of metabolic syndrome factors	0.111	0.453
Metabolic syndrome *z*-score	−0.253	0.082
Lifestyle factors		
Total energy intake (kJ/day)	−0.045	0.761
Carbohydrate intake (g/day)	0.352	0.013
Protein intake (g/day)	−0.177	0.225
Fat intake (g/day)	−0.157	0.283
Fibre intake (g/day)	0.393	0.006
Cardiorespiratory fitness		
Relative $\dot{V}$O_2peak_(mL/kg_FFM_/min)	0.294	0.042
Absolute $\dot{V}$O_2peak_(mL/min)	0.346	0.018
Smoking status ^2^	6.137 ^1^	0.046
Alcohol intake (drinks/week)	−0.226	0.056
Inflammatory marker		
HsCRP (mg/L)	−0.054	0.714
Glucose metabolism		
Fasting flucose (mmol/L)	−0.035	0.813
Fasting insulin (pmol/L)	0.079	0.594
HbA1c (%)	−0.074	0.670
HOMA-IR	−0.001	0.994
HOMA-B	0.113	0.444
pro-insulin (pM)	0.142	0.342
C-peptide (pmol/L)	0.043	0.771
Lipid profile		
Total cholesterol (mmol/L)	0.001	0.993
HDL-C (mmol/L)	−0.378	0.008
LDL-C (mmol/L)	0.114	0.452
Triglycerides (mmol/L)	−0.196	0.183

^1^ For categorical variables, Mann–Whitney test results are reported. ^2^ Smoking status: never smoker, ex-smoker, or current smoker. BMI: body mass index; MetS: metabolic syndrome; IL-22: interleukin-22; HsCRP: high-sensitivity c-reactive protein; HbA1c: glycated haemoglobin; HOMA-IR: homeostasis model assessment for insulin resistance; HOMA-B: homeostasis model assessment for insulin secretion; HDL-C: high-density lipoprotein cholesterol; LDL-C: low-density lipoprotein cholesterol; $\dot{V}$O_2peak_: peak oxygen uptake; FFM: fat-free mass.

**Table 3:** Hierarchical multiple regression coefficients for the variables included in each model (circulating IL-22 levels as the dependent variable).

Model Variable	Model 1		Model 2	
	Standardised β	*p*-Value	Standardised β	*p*-Value
WHR	0.544 (1.127–18.557)	0.028	0.607 (2.667–19.270)	0.011
HDL-C (mmol/L)	−0.025 (−3.045–−2.676)	0.897	−0.105 (−3.542–1.969)	0.558
Smoking	−0.315 (−1.694–−0.109)	0.027	−0.239 (−1.457–0.42)	0.081
Lean body mass (g)	0.388 (−1.547–7.132)	0.201	0.529 (−0.389–8.005)	0.074
$\dot{V}$O_2peak_(mL/kg_FFM_/min)	0.201(−0.026–0.120)	0.201	0.084 (−0.053–0.092)	0.467
Age (years)	−0.026 (−0.051–0.042)	0.856	−0.013 (−0.046–0.042)	0.924
Sex	−0.620 (−4.025–0.063)	0.057	−0.895 (−4.932–−0.786)	0.008
Fibre (g/day)	--	--	0.361(0.223–2.689)	0.022
**Model summary**	**Model 1**		**Model 2**	
R^2^	0.339		0.423	
R^2^ change	--		0.084	
*p*-value	0.014		0.022	

BMI: body mass index; MetS: metabolic syndrome; IL-22: interleukin-22; WHR: waist-to-hip ratio; HDL-C: high-density lipoprotein cholesterol; $\dot{V}$O_2peak_: peak oxygen uptake; FFM: fat-free mass.

## Supplementary Materials

The following are available online at https://www.mdpi.com/2072-6643/11/4/815/s1, Figure S1: Bi-variate correlation between circulating IL-22 and Metabolic Syndrome *z*-score, Table S1: Comparisons between low-fibre vs. high-fibre intake groups.
