# Supplementary material for: Fibre Intake Is Independently Associated with Increased Circulating Interleukin-22 in Individuals with Metabolic Syndrome
Source: Nutrients. 2019 Apr 11;11(4):815. doi: 10.3390/nu11040815 (PMC6520738; doi:10.3390/nu11040815)
Supplement: Supplementary file 1 [file nutrients-11-00815-s001.pdf]

Table S1 – Comparisons between low- vs high-fibre intake groups

**Variable**	**Low-Fibre (<21.5 g/day) n=24**	**High-Fibre (>21.5g/day) n=24**	**p-value**
Age (years)	57.7±7.7	55.6±11.0	0.461
Male %	54.2	70.8	0.556
MetS factors (>4) %	62.5	75.0	0.350
Metabolic Syndrome z-score	4.8±2.6	4.3±2.6	0.549
Body fat %	**41.3±6.7**	**37.1±7.0**	**0.024**
Average SBP (mmHg)	129.8±14.5	134.7±14.0	0.248
Average DBP (mmHg)	**81.4±7.3**	**87.2±10.5**	**0.038**
Waist-to-hip ratio	0.92±0.1	0.94±0.1	0.771
Mean OGTT glucose(mg/dl)	**263.5±72.7**	**185.3±63.0**	**0.027**
Type 2 Diabetes %	41.7	41.7	1.000
Aspirin use %	**33.3**	**8.3**	**0.033**
Never smoked %	**25.0**	**79.2**	**0.001**
Fibre intake (g/day)	**16.1±3.0**	**28.9±7.7**	**0.001**
CHO intake (g/day)	**164.3±53.1**	**224.0±80.4**	**0.004**
Energy (Kcal/day)	1847.7±737.8	2163.1±550.8	*0.101*
Cardio-respiratory fitness ($\dot{V}O_{2peak}$ – mL/kg/min)	25.7±5.9	29.9±7.1	*0.064*
IL-22 (pg/ml)	**69.0±106.4**	**308.3±454.4**	**0.019**
HsCRP (mg/l)	2.7 ±2.5	2.3±3.1	0.716

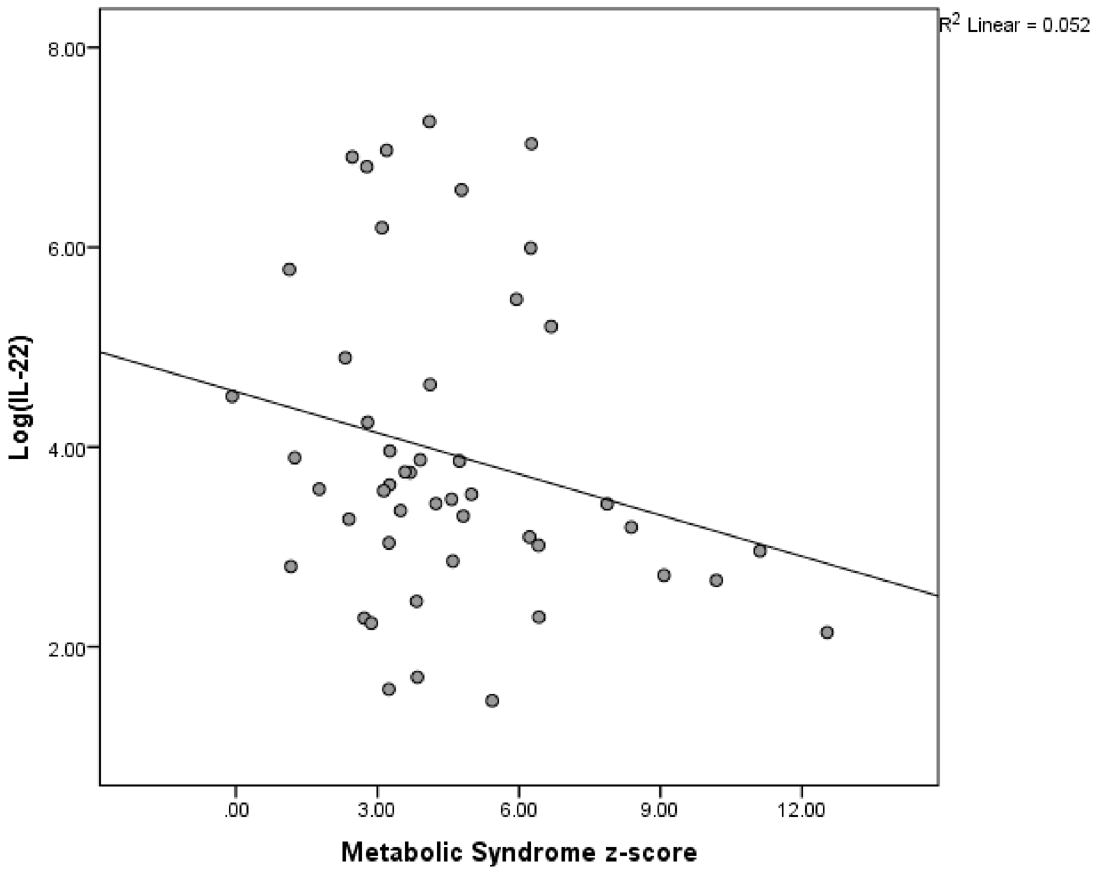

Figure S1. Bi-variate correlation between circulating IL-22 and Metabolic Syndrome z-score
